# Emergence of Minor Drug-Resistant HIV-1 Variants after Triple Antiretroviral Prophylaxis for Prevention of Vertical HIV-1 Transmission

**DOI:** 10.1371/journal.pone.0032055

**Published:** 2012-02-23

**Authors:** Andrea Hauser, Julius Sewangi, Paulina Mbezi, Festo Dugange, Inga Lau, Judith Ziske, Stefanie Theuring, Claudia Kuecherer, Gundel Harms, Andrea Kunz

**Affiliations:** 1 Institute of Tropical Medicine and International Health, Charité – Universitätsmedizin Berlin, Berlin, Germany; 2 Center for HIV and Retrovirology, Robert Koch-Institute, Berlin, Germany; 3 Regional AIDS Control Program Mbeya Region, Ministry of Health and Social Welfare, Dar es Salaam, Tanzania; 4 PMTCT Service Mbeya Region, Ministry of Health and Social Welfare, Dar es Salaam, Tanzania; 5 Kyela District Hospital, Ministry of Health and Social Welfare, Dar es Salaam, Tanzania; INSERM, France

## Abstract

**Background:**

WHO-guidelines for prevention of mother-to-child transmission of HIV-1 in resource-limited settings recommend complex maternal antiretroviral prophylaxis comprising antenatal zidovudine (AZT), nevirapine single-dose (NVP-SD) at labor onset and AZT/lamivudine (3TC) during labor and one week postpartum. Data on resistance development selected by this regimen is not available. We therefore analyzed the emergence of minor drug-resistant HIV-1 variants in Tanzanian women following complex prophylaxis.

**Method:**

1395 pregnant women were tested for HIV-1 at Kyela District Hospital, Tanzania. 87/202 HIV-positive women started complex prophylaxis. Blood samples were collected before start of prophylaxis, at birth and 1–2, 4–6 and 12–16 weeks postpartum. Allele-specific real-time PCR assays specific for HIV-1 subtypes A, C and D were developed and applied on samples of mothers and their vertically infected infants to quantify key resistance mutations of AZT (K70R/T215Y/T215F), NVP (K103N/Y181C) and 3TC (M184V) at detection limits of <1%.

**Results:**

50/87 HIV-infected women having started complex prophylaxis were eligible for the study. All women took AZT with a median duration of 53 days (IQR 39–64); all women ingested NVP-SD, 86% took 3TC. HIV-1 resistance mutations were detected in 20/50 (40%) women, of which 70% displayed minority species. Variants with AZT-resistance mutations were found in 11/50 (22%), NVP-resistant variants in 9/50 (18%) and 3TC-resistant variants in 4/50 women (8%). Three women harbored resistant HIV-1 against more than one drug. 49/50 infants, including the seven vertically HIV-infected were breastfed, 3/7 infants exhibited drug-resistant virus.

**Conclusion:**

Complex prophylaxis resulted in lower levels of NVP-selected resistance as compared to NVP-SD, but AZT-resistant HIV-1 emerged in a substantial proportion of women. Starting AZT in pregnancy week 14 instead of 28 as recommended by the current WHO-guidelines may further increase the frequency of AZT-resistance mutations. Given its impact on HIV-transmission rate and drug-resistance development, HAART for all HIV-positive pregnant women should be considered.

## Introduction

Mother-to-child transmission of HIV-1 in resource-limited settings accounts for almost 16% of all new HIV-1 infections in Sub-Saharan Africa [1]. Antiretroviral drugs for HIV-1-infected pregnant women and their infants are an essential component in reducing mother-to-child transmission of HIV-1. The non-nucleoside reverse transcriptase inhibitor (NNRTI) nevirapine (NVP) has been widely applied as single dose (NVP-SD) prophylaxis at the onset of labor [2]. However, due to the low genetic barrier of NVP even a single dose frequently induces viral resistance [3]–[10], thus compromising the success of subsequent NNRTI-containing highly active antiretroviral treatment (HAART) if initiated within 6–12 month after prophylaxis [11]–[13]. To reduce viral resistance as well as to further lower the vertical transmission risk of HIV-1, the WHO guidelines for the prevention of mother-to-child transmission (PMTCT) of 2006 and 2010 [14], [15] recommend complex antiretroviral prophylaxis. This is composed of antenatal zivoduvine (AZT) for three (2006) or six months (2010), NVP-SD at labor onset and AZT/lamivudine (3TC) during labor and for one week postnatally. In 2008, complex prophylaxis was recommended by the national Tanzanian PMTCT guidelines as preferred PMTCT regimen [16]. Monotherapy of antiretroviral drugs, however, inherently involves the risk of drug resistance development. Selection of AZT-resistant virus during prenatal AZT monotherapy might decrease the efficacy of future AZT-containing prophylactic and therapeutic regimens. Furthermore, as both NVP and 3TC rapidly select for drug-resistant virus, dual- or multi-resistant HIV-1 variants could emerge. Even minor drug-resistant HIV-1 variants representing small proportions of the total viral population can impair virological outcome of HAART [17]–[24]. Hence, it is mandatory to characterize the resistance development including minority species following complex prophylaxis, which to our knowledge has not been assessed for the WHO-recommended complex prophylaxis regimen. The aim of this study was to evaluate the emergence of HIV-1 variants resistant against AZT, NVP and/or 3TC following complex antiretroviral prophylaxis in a rural district hospital in Kyela, Mbeya Region, Tanzania. For this purpose, we developed, evaluated and applied highly sensitive allele-specific PCR (ASPCR) assays enabling the detection and quantification of three key mutations for AZT resistance (K70R, T215Y and T215F), the two most common NVP-associated resistance mutations (K103N and Y181C) and the most frequent 3TC-selected mutation M184V in the *pol* open reading frame with a detection limit of <1% [25], [26]. ASPCR assays were adapted for HIV-1 subtypes A, C and D which are common in Sub-Saharan Africa and prevalent in Mbeya Region, Tanzania [27]. Subsequently, blood specimens from HIV-1-infected pregnant Tanzanian women and their vertically infected infants who had taken complex antiretroviral prophylaxis were analyzed.

## Materials and Methods

### Ethics Statement

Ethical approval was obtained from the local Mbeya Medical Research and Ethics Committee, the National Institute for Medical Research of Tanzania and the ethical committee of Charité – Universitätsmedizin Berlin in Germany. We obtained informed written consent from all participants involved in our study.

### Clinical samples and study design

The present study analyzes the HIV-1 resistance development in HIV-1-infected Tanzanian women and their infants as part of an observational study at Kyela District Hospital, Mbeya Region between October 2008 and September 2009 [28]. In March 2008, complex antiretroviral prophylaxis was introduced as the standard PMTCT regimen at Kyela District Hospital. According to WHO PMTCT guidelines from 2006 [14] and National Tanzanian PMTCT guidelines [16], women were offered complex antiretroviral prophylaxis composed of AZT starting in gestational week 28 (2×300 mg per day), or as soon as possible thereafter, followed by NVP-SD (200 mg) at labor onset and AZT (300 mg) every three hours plus 3TC (150 mg) every 12 hours during labor, followed by a one week postpartum course of AZT (2×300 mg per day) and 3TC (2×150 mg per day). Infants received NVP-SD (2 mg/kg) within 72 hrs after birth and AZT (4 mg/kg per day) for one week. In case the mother had taken antenatal AZT for less than four weeks, the infant received postnatal AZT for four weeks. Blood samples were collected before start of AZT prophylaxis, during pregnancy, at delivery and at 1–2, 4–6, and 12–16 weeks postnatally.

202 of 1395 (14.5%) pregnant women tested for HIV-1 during antenatal care were HIV-1 positive. 122 HIV-positive women were included in the observational study as they fulfilled the following eligibility criteria: no HAART, no clinical or immunological indication to start HAART, i.e. CD4 cell count > = 200 cells/mm3 and clinical categories A or B according to CDC classification, age > = 18 years, absence of other severe diseases including psychiatric disorders, written informed consent [28]. Eventually, 87 of the 122 eligible women started AZT prophylaxis during pregnancy [28]. Women and if applicable their HIV-infected infants were included in the resistance analysis if they had taken AZT in pregnancy for at least two weeks, if they had taken NVP at labor onset, and if a delivery sample and at least two postnatal (1–2 weeks, 4–6 weeks and/or 12–16 weeks) plasma samples were available. In the case of home delivery, the last antenatal specimen was used as “delivery sample”. Additionally, a baseline sample prior to AZT intake had to be amplifiable in order to establish an individual cut-off for resistance detection [29]. No woman received any other antiretroviral drugs during the study period. Children of the study cohort were breastfed.

### Detection and quantification of drug-resistant HIV-1

Drug-resistant mutations in the *pol* open reading frame of HIV-1 were detected by ASPCR which is an established and widely used method for the analysis of minor drug-resistant HIV-1 variants [5], [29]–[33]. The assay is composed of two consecutive real-time PCRs. The outer real-time PCR amplified a reverse transcriptase (RT) fragment comprising the codons of interest (codons 22 to 236 of the RT) and was also used for quantification of viral load. The inner ASPCR was composed of one real-time PCR reaction with discriminatory ability for mutant sequences using selective primers and one generic real-time PCR reaction amplifying both wild-type and mutant sequences using non-selective primers (Table 1). For each resistance mutation, an individual inner ASPCR assay had to be designed. In total, seven ASPCR assays were performed per sample: two AZT mutations confering high level resistance (T215Y, T215F) and one early AZT mutation (K70R) confering only low level resistance but indicating for emergence of AZT-resistance; additionally the two most common NVP-selected resistance mutations (K103N and Y181C) and the most frequent 3TC-selected mutation M184V were analysed [34], [35] (details in Materials and Methods S1).

**Table 1:** Oligonucleotide sequences of primers used in outer and allele-specific PCR (ASPCR).

Assay and primer name	Nucleotide sequence	Nucleotide position (HXB2)	Fragment size (bp)
Outer-PCR			
HIV-TZ FOR	5′- AAACAATGGCCATTRACAGARGA-3′+	2613–2635	
HIV-TZ REV	5′- GGATGGAGTTCATAICCCATCCA-3′−	3234–3256	644
K70R ASPCR			
TZ-K70 FOR 1	5′- GCIATAAARAARAARGACAGYACTC-3′+	2733–2757	
TZ-K70R FOR 2	5′- GCIATAAARAARAARGACAGYACTCG-3′+	2733–2758	
TZ-K70 REV	5′- CCCACATCYAGTACTGTYACTGATTT-3′−	2859–2884	152
K103N ASPCR			
TZ-K103 FOR	5′- GGCCTGAAAATCCATAYAAYACTCC-3′+	2701–2725	
TZ-K103 REV1	5′- CCCACATCYAGTACTGTYACTGATTT-3′−	2859–2884	
TZ-K103N(C) REV3	5′- CCCACATCYAGTACTGTYACTGATTGG-3′−	2858–2884	
TZ-K103N(T) REV4	5′- CCCACATCYAGTACTGTYACTGATTGA-3′−	2858–2884	184
Y181C ASPCR			
TZ-Y181/M184 FOR	5′- AAATCAGTRACAGTACTRGATGTRGG-3′+	2859–2884	
TZ-Y181 REV1	5′- ATCCTACATACAARTCATCCATRTATTGA-3′−	3092–3120	
TZ-Y181C REV3	5′- ATCCTACATACAARTCATCCATRTATTGCC-3′−	3091–3120	262
M184V ASPCR			
TZ-Y181/M184 FOR	5′- AAATCAGTRACAGTACTRGATGTRGG-3′+	2859–2884	
TZ-M184 REV1	5′- TCAGATCCTACATAYAARTCATCCA-3′−	3101–3124	
TZ-M184V REV3	5′- TCAGATCCTACATAYAARTCATCIGC-3′−	3098–3124	266
T215Y/F ASPCR			
TZ-T215 FOR	5′- CACAGGGATGGAAAGGATCACC-3′+	2998–3019	
TZ-T215 REV1	5′- CTTCTGATGYTTYTTGTCTGGIGT-3′−	3185–3205	
TZ-T215Y REV3	5′- CTGATGYTTYTTGTCTGGIGTCTA-3′−	3182–3205	
TZ-T215F REV4	5′- CTGATGYTTYTTGTCTGGIGTCAA-3′−	3182–3205	
TZ-T215F REV5	5′- CTGATGYTTYTTGTCTGGIGTTAA-3′−	3182–3205	208

### Vertical transmission of HIV-1

The HIV-status of newborns was determined by RT-PCR of blood specimens collected 4–6 weeks after birth using the above described outer PCR. Infants with a positive PCR result at 4–6 week were defined to be HIV-infected whereas infants with a negative PCR result were assumed to be not HIV-infected. If the 4–6 week sample was lacking, an earlier blood sample from delivery or week 1–2 was analysed. If the earlier sample was PCR-positive, the child was considered to be HIV-infected 4–6 weeks after birth as well; if the earlier blood sample was PCR-negative, the infant was excluded from calculation of transmission rate as the HIV status week 4–6 after birth could not be determined.

### Population-based sequencing and determination of HIV-1 subtype

For population-based sequencing of the 644 bp product generated by outer PCR, the automated sequencer 3130xl Genetic Analyzer (Applied Biosystems, Darmstadt, Germany) and the HIV SEQ MIX B, D and G of the Viroseq HIV-1 Genotyping System version 2.0 (Abbott, Wiesbaden, Germany) were applied. To exclude sample mix-up and to confirm vertical HIV-1 transmission, phylogenetic analysis of maternal and infant sequences generated by population-based sequencing was performed using the neighbor joining method (Bioedit 7.0.9) [36]. HIV-1 subtyping of the *pol* sequence was performed using the REGA HIV-1 subtyping tool [37].

### Statistical analysis

The non-parametric Mann-Whitney U test was used to assess significant differences between two independent samples whereas the Wilcoxon signed-rank test was used to analyze repeated measurements. Chi-Square test or Fisher's exact test were applied to analyze the independence of categorical variables. Testing of significant correlations between two continuous variables was done by Pearson's correlation coefficient. For descriptive analysis, median and interquartile ranges (IQR) were calculated. Two-sided tests were used and p<0.05 was considered statistically significant. Drug-resistant HIV-1 variants carrying the K103N (AAC) mutation and the K103N (AAT) mutation were summed to obtain the total proportion of virus carrying the K103N mutation. Statistical analysis was carried out using PASW Statistics 18 (SPSS Inc., Chicago, Illinois, USA).

## Results

### Sample characteristics

Of 87 women having started complex prophylaxis, 50 women fulfilled the eligibility criteria and were included in the resistance analysis, together with their seven vertically HIV-infected infants. Median baseline characteristics before start of prophylaxis were: age 28 years (IQR 26–30), HIV-1 viral load 1.25×10^4^ copies/mL (IQR 4.4×10^3^–4.5×10^4^) and CD4 cell counts of 390 cells/mm^3^ (IQR 260–492). The median maternal viral load was 2.9×10^3^ copies/mL (IQR 1.4×10^3^–6.8×10^3^) at delivery, 1.7×10^3^ copies/mL (IQR 1.3×10^3^–5.8×10^3^) 1–2 weeks postpartum, 1.2×10^4^ copies/mL (IQR 6.3×10^3^–3.7×10^4^) 4–6 weeks postpartum and 2.5×10^4^ copies/mL (IQR 1.2×10^4^–3.7×10^4^) 12–16 weeks postpartum. Compared to baseline viral load, maternal viral loads at delivery and 1–2 weeks postpartum were significantly lower (both p<0.001) but reached similar levels at 4–6 weeks (p = 0.45) and at 12–16 weeks (p = 0.54) postpartum, respectively. Women received AZT during pregnancy for a median of 53 days (IQR 39–64). Thirty-seven (74%) women delivered at Kyela District Hospital whereas 13 (26%) women delivered at home or in another health facility. Regardless of the place of delivery, all women took NVP-SD before birth. Thirty-four of 37 women who delivered at Kyela District Hospital received intrapartum AZT/3TC. Forty-one women took AZT/3TC postpartum for one week, while another five women took AZT but not 3TC postpartum. In total, 86% (43/50) of women took at least one dose of 3TC. Forty-four (88%) infants received NVP-SD after birth, including all 37 newborns born at Kyela District Hospital and 7/13 infants born at another place. Forty-five (90%) newborns took AZT postnatally; 42 of whom for one week and three for four weeks. Forty-nine of 50 infants including all HIV-infected infants were breastfed. 28% (14/50) of the women were infected with HIV-1 subtype A1, 68% (34/50) with subtype C and two women (4%) with subtype D. None of the 50 baseline samples exhibited preexisting drug-selected mutations in the RT as determined by population sequencing.

### Quantification of HIV-1 RNA by outer PCR

A standard curve was calculated from eight independent runs (r^2^ = 0.992, standard deviation 0.004) by using defined concentrations of HIV-1 NL4.3 virus ranging from 6.5×10^1^–10^7^ copies/ml (details in Materials and Methods S1). The lower limit of detection for HIV-1 RNA was 650 copies/ml.

226 maternal samples (mean 4.5 samples per woman) were available, of which 211 were successfully amplified and quantified in the outer PCR, including 50/50 baseline samples, 48/50 delivery samples, 37/46 1–2 weeks samples (which displayed the lowest viral load), 47/49 4–6 weeks samples and 29/31 12–16 weeks samples. Out of the seven vertically HIV-1-infected newborns, 11/15 available samples were amplifiable in the outer PCR.

### Evaluation of ASPCR assays

#### Accuracy, precision, sensitivity and specificity of ASPCR

Accuracy, precision and sensitivity (detection limit) of all ASPCR assays are shown in Table 2. The coefficient of variation as measurement of inter-assay precision did not exceed 47% (range 12%–47%, data not shown). The lower detection limit for evidence of minor drug-resistant HIV-1 variants was 0.99% for K70R, 0.04% for K103N (AAC), 0.01% for K103N (AAT), 0.35% for Y181C, 0.63% for M184V, 0.33% for T215Y and 0.42% for T215F (Table 2). Specificity for HIV-1 wild-type controls was 100% for all ASPCR assays.

**Table 2:** Accuracy, inter-assay variability and detection limit of ASPCR assays to detect drug-resistant HIV-1 variants calculated from 7–9 independent experiments.

Input mutant allele (%)	Measured mean mutant allele (% ± standard deviation)													
	K70R (AGA)		K103N (AAC)		K103N (AAT)		Y181C (TGT)		M184V (GTG)		T215Y (TAC)		T215F (TTC)	
**100**	110	±33.6	115	±48.9	102	±20.4	108	±23.7	112	±24.5	116	±40.9	115	±31.5
**10.0**	9.35	±2.74	10.2	±2.59	10.9	±3.94	9.28	±2.72	8.38	±1.02	9.17	±2.63	11.7	±5.40
**1.00**	1.11	±0.42	0.85	±0.23	1.07	±0.42	1.12	±0.39	1.11	±0.22	1.09	±0.46	1.01	±0.47
**0.10**	0.29	±0.08	0.12	±0.05	0.10	±0.03	0.30	±0.08	0.27	±0.03	0.12	±0.06	0.11	±0.06
**0**	0.19	±0.08	0.01	±0.01	0.01	±0.01	0.08	±0.06	0.23	±0.03	0.05	±0.03	0.09	±0.04
**Detection limit (%)**	0.99		0.04		0.01		0.35		0.63		0.33		0.42	

Some maternal ASPCR results had to be excluded from analysis due to polymorphisms in primer binding sites (details in Materials and Methods S1); this affected two women for K103N analysis, one woman for Y181C analysis and six women for K70R analysis.

### Detection limit for drug-resistant HIV-1 in samples with low viral load

The sensitivity of ASPCR assays for detection of drug-resistant HIV-1 correlates with the input viral load. In order to avoid false positive results, we established a threshold considering the respective viral load of any given sample (see Materials and Methods S1). The lower detection limit for drug-resistant HIV-1 variants was 0.17% for samples with 10^4^ copies/ml and 0.97% for samples with 10^3^ copies/ml. If the calculated proportion of drug-resistant HIV-1 fell below the calculated threshold, it was considered to be false positive and presence of HIV-1 wild type was assumed; this affected the detection of K103N and T215Y only once.

### Emergence of drug-resistant HIV-1 variants in Tanzanian women

**Figure 1:**
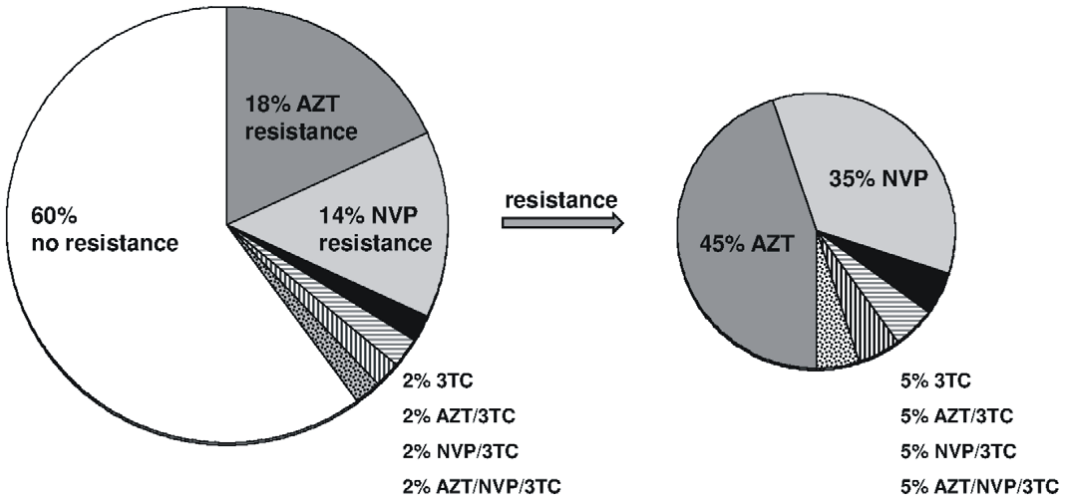
Distribution of drug-resistant HIV-1 variants after complex antiretroviral prophylaxis in 50 Tanzanian women.

In total, 20/50 (40%) women exhibited drug-resistant virus during the observation period (Table 3), including 13/34 (38%) women infected with HIV-1 subtype C, 6/14 (43%) women with subtype A1 and 1/2 with subtype D. Genotypic mutations associated with decreased susceptibility to AZT were detected in 11/50 (22%) women (7/50 (14%) containing K70R alone and 4/50 (8%) with T215Y/F mutation) whereas 9/50 (18%) women harbored NVP-resistant virus (K103N and/or Y181C). In 4/50 (8%) women a 3TC-resistance mutation (M184V) was identified, of these 3/50 (6%) developed drug-resistant HIV-1 strains against more than one drug (Figure 1).

**Table 3:** Drug-resistant HIV-1 variants in plasma samples of 20/50 women after complex antiretroviral prophylaxis as analyzed by allele-specific PCR (ASPCR).

No	Sub-type	Viral load delivery (cop/ml)	Antenatal AZT-intake (days)	Results of population sequencing and ASPCR							
				Delivery		Week 1–2		Weeks 4–6		Weeks 12–16	
				popseq	ASPCR	popseq	ASPCR	popseq	ASPCR	popseq	ASPCR
**1**	C	1,546	58	K70R	13% K70R		wt		-		wt
**2**	C	29,400	77	K70R	11% K70R	wt	0.7% M184V		wt		-
**3**	A1	97,450	28	K70R	14% K70R		wt	wt	5.4% K70R		-
**4**	A1	7,915	81	K70R	28% K70R		wt	K70R	14% K70R		wt
**5**	C	37,800	81	wt	2.0% K70R 0.5% T215F	K65R	0.5% T215F	wt	2.3% K70R	wt	0.7% T215F
**6**	A1	4,806	43		wt	wt	0.5%T215Y		wt		wt
**7**	A1	6,400	87		wt	wt	10% K103N	wt	0.8%Y181C		-
**8**	C	3,790	49		wt	wt	0.4% Y181C	wt	1.3% K103N		-
**9**	C	21,800	14		wt	wt	0.6% M184V	wt	3.4% K103N		wt
**10**	A1	3,455	95		wt		wt	wt	4.9% K70R		-
**11**	C	1,002	92		wt		wt	wt	2.7% K70R		-
**12**	C	1,079	33		wt		-	wt	0.8% T215F		wt
**13**	C	4,625	32		wt		wt	wt	3.9% T215Y		wt
**14**	A1	646	65		wt		wt	wt	2.1% K103N		-
**15**	C	2,150	67		wt		wt	wt	3.4% K103N		-
**16**	C	2,875	49		wt		wt	K103NY181CV106A	36% K103N 20% Y181C 0.6% M184V	K103N	12% K103N 4.0% K70R
**17**	D	1,480	48		wt		-		wt	wt	0.2% K103N
**18**	C	1,258	38		wt		-		wt	wt	0.4% Y181C
**19**	C	1,055	56		wt		wt		wt	G190A	1.5% Y181C
**20**	C	47,050	56		wt		wt		wt	wt	1.0% M184V

wt = wild-type HIV-1.

- = no sample/not amplifiable.

In 5/20 women, drug-resistant variants were already detectable at delivery and all of these women carried HIV-1 with AZT-selected resistance mutations only. In 4/20 women, resistant virus was detectable for the first time 1–2 weeks after delivery and in 11/20 women resistant variants were not present before weeks 4–6. 50% of the women with HIV-1 resistance still exhibited drug-resistant virus at week 12.

The first AZT-selected mutation emerging was the K70R, which was detectable at delivery in 5/50 women in proportions of 2%–28%. The shortest interval between the start of AZT prophylaxis and detection of the K70R mutation was 28 days (Table 3, no 3). T215Y and T215F mutations mostly emerged later and were measurable 1–6 weeks postpartum in 4/50 (8%) women in low proportions of 0.5%–3.9%. One woman displayed both AZT resistance mutations K70R and T215F in the viral genome, which were present already at delivery and persisted throughout the observation period at low frequencies (Table 3, no 5).

The total median viral load reduction from baseline to delivery was 0.6 log_10_; women with AZT-resistant virus at delivery displayed significantly lower reduction (0.1 log_10_) compared to women without AZT resistance at delivery (p = 0.045, Mann-Whitney U-test). Accordingly, women with AZT-resistant virus at delivery displayed significantly higher median viral load at delivery (29400 copies/ml) compared to women without AZT resistance at delivery (2680 copies/ml; p = 0.021, Mann-Whitney U-test). Furthermore, women exhibiting AZT-resistant virus at delivery had lower CD4 cell counts at baseline (331 cells/mm^3^) versus women without AZT resistance (406 cells/mm^3^); this difference marginally failed to reach statistical significance (p = 0.077, Mann-Whitney U-test).

The median number of days of antenatal AZT intake did not differ significantly between the five women who displayed AZT resistance mutation at delivery (77 days) and the 45 women without AZT-resistance at delivery (50 days; p = 0.20, Mann-Whitney U-test). However, the frequency of AZT resistance at delivery differed significantly in women with antenatal AZT intake of at least 10 weeks (3/10 = 33%) as compared to women who took antenatal AZT for less than 10 weeks (2/40 = 5%; p = 0.048, Fisher's exact test).

NVP resistance mutations K103N and/or Y181C were detected in postpartum samples of nine (18%) women, but the proportion of resistant variants never exceeded 5% during the study period in 7/9 (78%) of these women. In 2/9 (22%) women higher proportions were detectable (Table 3, nos. 7, 16). One of these women (no. 16) did take NVP-SD and AZT/3TC during labor, but did not receive the postpartum AZT/3TC-tail to avoid NVP-resistance development. This woman exhibited dual-resistant virus against NVP and 3TC at week 4–6 and dual-resistant virus against NVP and AZT at month three. 3/9 women who had not taken AZT and/or 3TC postpatum (Table 3, nos. 16, 17, 19) developed NVP-resistance compared to 6/41 women who took the postpartal tail correctly (p = 0.33, Fisher's exact test).

The 3TC-resistance mutation M184V was detected in four women (8%) in low proportions of 0.6%–1.0% and was no longer detectable in 3/4 women at week 12–16.

In 70% (14/20) of the women who developed drug-resistant HIV-1 variants the relative proportions of resistant populations never exceeded 5% during the whole study period. The range of proportions of drug-resistant HIV-1 variants was 0.2–36% for K103N mutants, 0.4–20% for Y181C mutants, 0.6–1.0% for M184V mutants, 2.0–28% for K70R mutants, 0.5–3.9% for T215Y mutants and 0.5–0.8% for T215F mutants, respectively. In total, 34 drug-resistant variants were detected; out of these, 12 were present in proportions <1%, 12 in proportions of 1–5%, and 10 in proportions of >5%.

Altogether, complex prophylaxis resulted in the development of drug resistance in 40% of HIV-infected women. Out of these, 45% carried HIV-1 with AZT-resistance mutations, 35% showed NVP single drug-resistance, 5% 3TC single drug-resistance and 15% dual or triple drug-resistance in the viral genome (Figure 1). A longer duration of antenatal AZT intake seemed to increase the risk for selection of AZT-resistance mutations. In most women drug-resistant virus was present as minority species only.

### Vertical transmission and emergence of drug-resistant HIV-1 variants in infected infants

Blood specimens collected 4–6 weeks after birth were available for 47/50 newborns; 5 were tested to be HIV-positive (no. 5, 6, 13, 21, 22; Table 4). In three additional cases, the 4–6 week sample was lacking, and an earlier sample (taken at delivery, 3 days or 2 weeks postpartum) was analyzed respectively: two of these samples were HIV-PCR positive, those infants were therefore assumend to be HIV-1 infected (no. 23, 24; Table 4). The third child was HIV-PCR negative, this infant was excluded from calculation of the transmission-rate. The overall HIV-transmission rate 4–6 weeks after birth was 14.3% (7/49 infants).

**Table 4:** Drug-resistant HIV-1 variants in plasma samples of seven children HIV-1 infected by vertical transmission as analyzed by allele-specific PCR (ASPCR).

No	Sub-type	Mother/child	Maternal CD4 count (cells/µl)	Maternal viral load (cop/ml)	Ante-natalAZT (days)	Drug intake during labor	Drug intake postnatal	Results of ASPCR			
								delivery	week 1–2	week 4–6	week 12–16
5	C	mother	344	37,800	81	NVP-SD	AZT/3TC	2.0% K70R° 0.5% T215F°	0.5% T215F°	2.3% K70R°	0.7% T215F°
		child					NVP-SD AZT	-	-	15% K70R * 3.4% K103N°	2.7% K70R°
6	A1	mother	572	4,806	43	NVP-SD AZT/3TC	AZT/3TC	wt	0.5%T215Y°	wt	wt
		child					NVP-SD AZT	n/a	-	wt	n/a
13	C	mother	678	4,625	32	NVP-SD AZT/3TC	AZT/3TC	wt	wt	3.9% T215Y°	wt
		child					NVP-SD AZT	wt	-	wt	wt
21	A1	mother	231	14,850	33	NVP-SD AZT	AZT/3TC	wt	wt	wt	-
		child					NVP-SD AZT	-	-	0.9% K103N° 2.5% Y181C°	-
22	C	mother	211	1,720	60	NVP-SD	-	wt	n/a	wt	-
		child					-	-	-	12% K103N° 12% Y181C°	-
23	C	mother	612	2,110	20	NVP-SD AZT/3TC	AZT/3TC	wt	wt	wt	wt
		child					NVP-SD AZT	n/a	wt	-	wt
24	A1	mother	200	5,385	46	NVP-SD AZT/3TC	AZT/3TC	wt	wt	wt	-
		child					NVP-SD AZT	n/a	wt #	-	-

wt = wild-type HIV-1.

n/a = not amplifiable.

- = no sample.

# = sample collected at day 3.

* = also detected by population-based sequencing.

° = not detected by population-based sequencing.

Vertical transmission was proven by phylogenetic analysis of maternal and infant HIV-1 sequences (data not shown). We did not observe a correlation between the vertical transmission risk of HIV-1 with either maternal CD4 cell count at enrolment, viral load at delivery or viral load reduction during pregnancy (p = 0.131; p = 0.388; p = 0.360, Mann-Whitney U-test) or with the presence of AZT-resistant HIV-1 variants (p = 0.546, Fisher's exact test). All children were at least exposed to maternal NVP-SD during delivery, and 44/50 (88%) infants took an additional dose of NVP postnatally. Eleven plasma samples of the seven HIV-infected infants were amplifiable in outer PCR and were available for subsequent ASPCR assays (Table 4). Three of 7 infants developed drug-resistant virus (Table 4, nos. 5, 21 and 22). Two infants (nos. 21 and 22) developed NVP-resistant HIV variants while both mothers exhibited wild-type virus only during the observation time. To one of these infants (no. 22) neither postnatal NVP nor AZT was administered, but the child developed high proportions of NVP-resistant virus at week 4–6. The third newborn (no. 5) carried resistant virus against AZT (K70R) and NVP (K103N) 4–6 weeks after birth; the mutation K70R was also detectable in the maternal delivery sample.

### Results of population-based sequencing and comparison with ASPCR results

Population-based sequencing was conducted on all maternal and infant samples with drug resistance mutations as determined by ASPCR (n = 34, Table 3 and Table 4) and additionally on 27 samples without indication of drug-resistant virus in the ASPCR (data not shown).

In all samples harboring resistant virus in proportions >20% according to ASPCR assays, population-based sequencing confirmed the presence of drug-resistant virus, and the presence of mutations as identified by population-based sequencing was always detected in the ASPCR assays (Table 3). All samples without detectable drug-resistant HIV-1 or with drug-resistant variants in proportions < = 10% in the ASPCR were identified to contain HIV wild-type only by population sequencing (Table 3).

We also checked population sequences for additional AZT/3TC/NVP-selected resistance mutations like M41L, D67N, K70R, L210W, T215Y/F and K219QE for AZT, K65R for 3TC and L100I, K101P, V106A/M, V108I, Y188C/L/H and G190A for NVP. Additional mutations in the HIV-1 genome were detected in three women: One woman each harbored the V106A (together with K103N, Y181C and M184V), the K65R (together with T215F) and the G190A (together with Y181C) mutation, respectively (Table 3, nos. 5, 16, 19).

## Discussion

Since 2006, WHO PMTCT guidelines recommend complex antiretroviral prophylaxis with AZT monotherapy during pregnancy, NVP-SD at labor onset, AZT/3TC during labor and for one week after delivery [14], [15]. Since AZT monotherapy and usage of drugs with low genetic barriers like NVP and 3TC might facilitate the formation of drug resistance, we aimed at monitoring the emergence and persistence of key resistance mutations selected by AZT, NVP and 3TC in 50 Tanzanian women from enrolment (before start of prophylaxis) up to three months postpartum. To our knowledge, this is the first study analyzing drug-resistance including minority species in women who had taken the WHO recommended complex prophylaxis.

### AZT resistance

Emergence of AZT-resistant virus after starting AZT monotherapy during pregnancy has been reported to be low with less than 3% occurrence [38], [39]. Applying our highly sensitive ASPCR assays capable of detecting minority species <1%, we detected HIV-1 with AZT-resistance mutations in a much higher proportion of women (11/50 = 22%). However, population-based sequencing, detecting minor variants in proportions only above 20%, revealed AZT-resistance mutations (K70R) in HIV-1 of only 4 women (8%). Furthermore, the women in our study displayed lower CD4 cell count levels (median: 390 cells/mm^3^) compared to the relatively immunocompetent women in other studies (median: >500 cells/mm^3^) [38], [39]. Advanced disease stage and low CD4 cell counts have been shown to be associated with a higher frequency of AZT-resistance [40], [41]. This is in accordance with our finding, that women carrying virus variants with AZT-selected mutations at delivery displayed a 10fold higher median viral load compared to women without AZT resistance mutation at delivery (p = 0.021, Mann-Whitney U-test). Furthermore, these women tended to display lower CD4 cell counts (median: 331 cells/mm^3^) in comparison to women without AZT resistance mutations (median: 406 cells/mm^3^; p = 0.077, Mann-Whitney U-test). In the most recent WHO guidelines (2010), AZT prophylaxis is recommended to start at a higher CD4 cell count level of 350 cells/mm^3^ instead of 200 cells/mm^3^ as in the previous 2006 guidelines. This might contribute to reduced emergence of AZT resistant HIV-1.

The shortest interval between start of AZT exposure and the emergence of AZT-selected mutation K70R was 28 days only. AZT resistance mutations were detected more frequently in HIV-1 of women who had taken AZT during pregnancy for longer than 10 weeks. In fact, in 30% of these women HIV carried AZT-resistance mutations at delivery. It is well known from other studies that the duration of AZT intake is associated with resistance development [40], [42], [43].

K70R was the most frequently observed AZT mutation in samples taken at delivery (n = 5), while T215Y and T215F mutations mostly emerged later during the observation period. In fact, the K70R mutation is considered to be an early AZT mutation and indicates the emergence of AZT-resistance followed by M41L, T215Y/F and L210W [34]. This might be due to the fact that for K70R one base substitution is sufficient (AAA/AAG to AGA/AGG) while for T215Y and F two base mutations are required (ACC to TAC = >T215Y or TTC = >T215F) [34]. 7/11 women with HIV-1 carrying AZT-selected mutants displayed the K70R mutation in proportions of 3%–28%, whereas T215Y/F-carrying virus was harbored in lower proportions of 0.5%–3.9% by four women. It is important to note that the K70R mutation affecting HIV-1 of 7/50 (14%) women confers low level resistance towards AZT, whereas T215Y and T215F mutations affecting virus of 4/50 (8%) women result in high-level resistance [34], [35]. While emergence of K70R is transient, AZT-resistant mutation T215Y is reported to persist for several months up to more than one year even after AZT discontinuation [44]–[46].

Antenatal AZT is supposed to reduce in-utero HIV-1 transmission. So far, it is not fully understood how exactly AZT is preventing in-utero transmission. Viral load reduction by AZT in pregnancy has been shown to be modest with −0.24 log_10_ and −0.3 log_10_ by Sperling [47] and Clarke [48] and with −0.6 log_10_ in our study. Therefore, since AZT readily crosses the placenta [49] it is rather conceivable that the child is at least also protected by pre- and post-exposure prophylaxis than by the maternal viral load reduction at delivery.

Since the AZT resistance mutation T215Y was shown to persist for several month [44]–[46], resistant variants could be re-selected if exposed to prophylactic AZT in future pregnancies or during subsequent AZT-containing HAART if initiated within this period after AZT exposure. This is of special importance for Sub-Saharan African populations as many women give birth to more than one child; AZT mutations may accumulate over time if AZT is used during consecutive pregnancies.

Our results are conflicting with the WHO statement that “the available evidence suggests that the time-limited use of AZT monotherapy during pregnancy for prophylaxis (for approximately six months, or less) should not be associated with a significant risk of developing AZT resistance” [15]. Compared to 2006, WHO guidelines from 2010 recommend to prepone the start of antenatal AZT to week 14 instead of week 28 [14], [15], corresponding to a 6-month AZT monotherapy. According to our findings, prolongation of antenatal AZT may increase the frequency of AZT-resistant virus.

### NVP and 3TC resistance

NVP-selected resistance mutations that cause cross-resistance to other NNRTIs are a major concern as NNRTIs are cornerstones of first-line HAART in resource-constrained settings. According to WHO guidelines, AZT/3TC should be taken by women for seven days postpartum to counteract the long presence of subtherapeutic NVP concentrations due to NVP's long half-life. NVP resistance was detected in 18% in our study group, which is a remarkable reduction compared to up to 87% after NVP-SD intervention [10]. The efficacy of postpartum short-course AZT/3TC-tails in reducing NNRTI resistance after intrapartum NVP-SD has indeed been shown in other studies [50], [51]. In our study group, 8% of women exhibited 3TC-resistant virus in very low proportions of <1% only. The M184V mutation results in complete resistance to 3TC and the presence of postpartum M184V in proportions >20% has been correlated to subsequent treatment failure using 3TC-containing HAART [52]. However, the clinical and virological relevance of 3TC-resistant virus in low proportions is not known. Moreover, M184V is known to be rapidly lost upon withdrawal of 3TC.

### Multiple drug resistance

In three women, resistant virus against more than one drug emerged during the observation period. The main risk factor for resistance development in general is incomplete adherence. The most severely affected woman with respect to HIV-1 resistance development (Table 3, no. 16) did not take AZT/3TC postpartum; it seems reasonable to assume that this fostered resistance development. It could be argued that the resistance development in this woman cannot be attributed to the effect of complex prophylaxis as it was not taken correctly. However, this might as well realistically reflect the existing conditions in rural settings and the challenges to adhere to a complex drug regimen.

### Minor drug resistance

In 70% (14/20) of the women with development of drug-resistant HIV-1, the resistant variants never exceeded proportions of 5%. The clinical relevance of these minority species is not fully understood and controversially discussed [17]–[24], [53]. There is evidence that minor drug-resistant variants can re-emerge in subsequent regimens leading to failure of salvage therapy [21]. While Metzner et al. [53] reported of successful treatment despite pre-existing minor K65R, K103N and M184V-variants in German Truvada cohort, several other studies have shown that the presence of drug-resistant minor variants increased the risk for subsequent treatment failure for NNRTI- [18]–[24], protease inhibitor- [17], [54], [55] and AZT-containing treatment [56]. While a single NNRTI-resistance mutation confers high-level resistance to some NNRTIs (an association with virologic failure in efavirenz-containing regimen was found for K103N variants at frequencies of > = 0.5% by Halvas et al. [57]), resistance to PI and AZT requires an accumulation of several mutations [58]. It is not yet fully understood at which threshold minor resistant viral populations may become clinically relevant. Furthermore, the threshold might be different for each resistance mutation and also depend on the subsequent treatment regimen. More evidence-based data are necessary to determine the role of minor drug-resistant HIV-1 in the response to antiretroviral therapy.

### Vertical transmission and emergence of drug-resistant HIV-1 variants in infected infants

The overall transmission rate in this study cohort of 50 mother-infant pairs 4–6 weeks after delivery was 14.3% and thus unexpectedly high. Neither a low CD4 cell count nor a high viral load at delivery in the transmitting mothers could be identified as transmission risk factors. Of 50 infants, all but one were breastfed, including all HIV-infected infants. We could not define the exact time of transmission for 4/7 infants due to lacking samples of delivery and/or of week 1–2. However, at least 3/7 children were born HIV uninfected (HIV-PCR was negative in the delivery sample). We therefore assume that postpartal transmission via breastmilk is the main reason for the high transmission rate.

Three of 7 infants developed drug-resistant HIV-1. In 2/3 newborns with NVP-resistant variants, mutations most likely emerged in the infants as both mothers exhibited wild-type HIV-1 only during the observation period. One infant, who did not take AZT and NVP postnatally (no. 22) exhibited NVP-resistant virus in high proportions at week 4–6 which was selected most likely by the maternal NVP dose. NVP rapidly crosses the placenta, resulting in high NVP concentrations in the infant's blood at birth [59], [60]. Postnatal NVP dosing of the infant only slightly elevated the NVP levels in infants [61]. Therefore an infant whose mother has taken NVP-SD during labor can develop NVP-resistant virus even without postnatal ingestion of NVP.

### Conclusions

Although complex antiretroviral prophylaxis decreased NVP-selected resistance compared to NVP-SD alone, HIV-1 with AZT-resistance mutations emerged in a substantial proportion of women. This may impact negatively future AZT-containing prophylaxis and HAART of the mother. In accordance with Katzenstein [62], we believe that it should be considered to substitute AZT monotherapy in pregnancy by HAART. There is growing evidence that starting HAART regardless of CD4 cell count level is highly beneficial for all HIV-infected individuals [63]–[66]. Additionally, HAART during pregnancy seems to be safe and advantageous for maternal and infant health [67]–[70] although it is important to further monitor the long-term effects of antiretroviral drugs on HIV-exposed but uninfected children [71]. In the light of the accumulating knowledge on the detrimental nature of untreated HIV-1, it seems justified to treat this infectious disease as soon as it is diagnosed instead of delaying medication until destructions of immune functions have taken place. Therefore, we advocate for HAART for *all* HIV-positive pregnant women; this equals “option B” in WHO guidelines of 2010 [15]. However, beyond that HAART should be considered lifelong and not be stopped after delivery, as discontinuation increases the risk of future treatment failure when restarting HAART [72]. This approach would minimize the risk of HIV-1 transmission and of resistance development, would allow breastfeeding and have an overall beneficial impact on HIV-1-infected mothers and their children.

## Supporting Information

Materials and Methods S1 (DOC)
