# Supplementary material for: Emergence of Minor Drug-Resistant HIV-1 Variants after Triple Antiretroviral Prophylaxis for Prevention of Vertical HIV-1 Transmission
Source: PLoS One. 2012 Feb 23;7(2):e32055. doi: 10.1371/journal.pone.0032055 (PMC3285650; doi:10.1371/journal.pone.0032055)
Supplement: Materials and Methods S1 — (DOC) [file pone.0032055.s001.doc]

#### PLoS ONE: Supplementary materials and methods S1

“Emergence of Minor Drug-resistant HIV-1 Variants after Triple Antiretroviral Prophylaxis for Prevention of Vertical HIV-1 Transmission”

**Emergence of Minor Drug-resistant HIV-1 Variants after Triple Antiretroviral Prophylaxis for Prevention of Vertical HIV-1 Transmission**

Andrea Hauser^1,2^*, Julius Sewangi^3^, Paulina Mbezi^4^, Festo Dugange^5^, Inga Lau^1^, Judith Ziske^1^, Stefanie Theuring^1^, Claudia Kuecherer^2^, Gundel Harms^1^, Andrea Kunz^1^

## 1 Institute of Tropical Medicine and International Health, Charité – Universitätsmedizin Berlin, Berlin, Germany

## 2 Center for HIV and Retrovirology, Robert Koch-Institute, Berlin, Germany

## 3 Regional AIDS Control Program Mbeya Region, Ministry of Health and Social Welfare, Dar es Salaam, Tanzania

## 4 PMTCT Service Mbeya Region, Ministry of Health and Social Welfare, Dar es Salaam, Tanzania

## 5 Kyela District Hospital, Ministry of Health and Social Welfare, Dar es Salaam, Tanzania

***E-mail:** [**andrea.hauser@charite.de**](mailto:andrea.hauser@charite.de)**, hausera@rki.de**

This PDF file includes:

Supplementary Materials and Methods S1

References for Supplementary Materials and Methods S1

**Supplementary Materials and Methods S1**

#### *Cloned DNA standards*

DNA plasmid standards carrying the K70R (AGA), K103N (AAC or AAT) and Y181C (TGT) mutation in the RT and the sensitive wild-type control were generated as described previously [1]. Briefly, PCR-amplified HIV-1 *pol*-fragments (nt 2001 to 3454 of HXB2; Acc. No. [K03455](http://www.ncbi.nlm.nih.gov/sites/entrez?cmd=Retrieve&db=nucleotide&dopt=GenBank&list_uids=1906382)) from patients with resistant HIV-1 and wild-type sequences (pNL4-3 WT, Acc. No. M19921) were cloned into plasmid pBluescript II SK(+) (pBSSK+, pBlueskript; Stratagene GMBH, Heidelberg, Germany). DNA plasmids carrying the M184V (GTG), T215Y (TAC) and T215F (TTC) mutation in the RT were generated by mutagenesis of wild-type plasmid DNA with QuikChange® II Site-Directed Mutagenesis Kit according to the manufacturer’s instructions (Stratagene GmbH, Heidelberg, Germany). Primers for mutagenesis were designed with the primer-design programme PrimerX [2].

MG_M184_F: GTCATCTATCAATACGTGGATGATTTGTATG;

MG_M184_R: CATACAAATCATCCACGTATTGATAGAATGAC;

MG_T215Y_F: GTTGAGGTGGGGATTTTACACACCAGACAAAAAAC;

MG_T215Y_R: GTTTTTTGTCTGGTGTGTAAAATCCCCACCTCAAC;

MG_T215F_F: GTTGAGGTGGGGATTTTTCACACCAGACAAAAAAC;

MG_T215F_R: GTTTTTTGTCTGGTGTGAAAAATCCCCACCTCAAC.

The mutant sequence of each clone was verified by Sanger sequencing.

Standard curves for quantification of drug-resistant virus were established using mixtures of DNA standards carrying the mutant codon (AGA at codon 70, AAC or AAT at codon 103, TGT at codon 181, GTG at codon 184 or either TAC or TTC at codon 215) with wild-type DNA standards (AAA at codon 70, AAA at codon 103, TAT at codon 181, ATG at codon 184 or ACC at codon 215) at defined ratios (0%, 0.1%, 1%, 10% and 100% proportion of mutant amplicons; in total 10^8^ copies/ml). Seven to nine runs were performed independently and the means were calculated and used for subsequent analysis.

#### *RNA standards*

The true detection limit for minor drug-resistant HIV-1 in clinical samples depends on the total viral load and can be much lower than the detection limit derived from DNA standards at high concentrations. Since most of our clinical samples exhibited relatively low viral loads, we determined the detection limit by analyzing RNA controls ranging from 10^3^ to 10^5^ cop/ml. This approach also takes into account that reverse transcription never results in 100% reverse-transcribed RNA templates.

For this purpose, two RNA standards carrying either T215T (ACC) or T215Y (TAC) codons were obtained from RNA extraction of recombinant HIV-1 strains produced in cell culture. To generate recombinant virus, cloning was performed as described by Walter et al. [3]. Briefly, *pol*-fragments from selected pBSSK^+^ clones were reamplified by PCR and cloned into the pNL4-3ΔPRT5 *ApaI* and *NheI/XbaI* restriction sites, resulting in plasmid DNA containing full-length recombinant HIV genomes. Clonal plasmid DNA was transfected into HEK293T cells (Cell Line 293T/17; American Type Culture Collection, Manassas, USA) using the SuperFect Transfection Reagent (QIAGEN GmbH, Hilden, Germany) according to the manufacturer’s instructions. Two days after transfection, cleared supernatant was harvested and stored at -70°C. Population sequencing of the *pol* region (nt 2001 to 3454 of HXB2; Acc. No. [K03455](http://www.ncbi.nlm.nih.gov/sites/entrez?cmd=Retrieve&db=nucleotide&dopt=GenBank&list_uids=1906382)) was performed to verify the presence of wild-type and mutant codons in the respective virus stocks.

The HIV-1 wild type and T215Y mutant RNA were quantified and diluted to concentrations ranging from 10^3^ to 10^5^ cop/ml and then mixed in different ratios: 100%, 10%, 5%, 2.5%, 1.25%, 1%, 0.5%, 0.1% and 0.01% mutant amplicons for RNA standard with a total concentration of 10^3^ copies/ml; 100%, 10%, 5%, 2.5%, 1.25%, 1%, 0.5%, 0.25%, 0.125%, 0.1% and 0.01% mutant amplicons for RNA standard with a total concentration of 10^4^ copies/ml; and 100%, 10%, 1%, 0.1% and 0.01% mutant amplicons for RNA standard with a total concentration of 10^5^copies/ml, respectively. Four independent serial mixtures for each of the three RNA concentrations (10^3^, 10^4^, 10^5^ cop/ml) were performed and measured in duplicate. The lowest mutant proportion which was still detectable in all four serial mixtures was determined for each RNA concentration and used to calculate a standard curve. The detection threshold for any specific viral load was deduced from the standard curve.

#### *Primer design for outer real-time PCR and inner ASPCR*

Primers for the outer PCR were specifically designed for HIV-1 subtypes A, C and D by aligning the reference sequence panel of the Los Alamos HIV-1 sequence database [4] together with a panel of published Tanzanian HIV-1 sequences collected in the study region of Mbeya [5-7] (Table 1). The outer 644-bp PCR fragment of all clinical baseline samples were analyzed by population-based sequencing, and the sequences obtained were taken into account for the primer of the inner ASPCR (Table 1). To counterbalance HIV-1 subtype-specific differences and polymorphisms in the primer-binding sites, wobbled bases and/or Inosine were introduced in some primers (Table 1). An additional selective primer considering polymorphisms in the primer-binding region was designed for T215F detection in some samples. Primer-binding sites of DNA standards were matched accurately to the primers used in outer and inner ASPCR.

#### *Outer PCR and quantification of HIV-1 RNA*

Outer PCR was conducted as described previously [1] with the following slight changes: After reverse transcription of 10 μl RNA (100 μl plasma equivalents), outer real-time PCR was performed using 0.75 μM each of forward primer HIV-TZ FOR and reverse primer HIV-TZ REV ([Table 1](http://www.ncbi.nlm.nih.gov/pmc/articles/PMC2704644/table/t1/)).

For quantification of HIV-1 RNA viral load in maternal and newborn samples, a standard curve for each run was calculated using serial dilutions of HIV-1 negative human plasma spiked with a defined amount of HIV-1 NL4.3 virus (NL4-3 WT, Acc. No. M19921) ranging from 6.5 x 10^1^ - 10^7^ copies/ml. All samples were run in duplicate and the mean was used for subsequent analysis.

*ASPCR assays and their validation*

For each resistance mutation to be analyzed, a specific ASPCR assay was developed separately. Each ASPCR assay was composed of two real-time PCRs: one to amplify mutant sequences only using mutant-specific primers, and one generic reaction to amplify both wild-type and mutant sequences using non-selective primers (Table 1). Both generic and mutant-specific real-time PCRs were performed separately and the threshold cycles/crossing points (CPs) were used for calculation of ΔCP (CP of mutant-specific PCR minus CP of generic PCR). ASPCR assays were performed as previously described [1] by using 2 µl of the outer PCR product (1:200 diluted in 5 mM Tris-HCl buffer, pH 8). All ASPCR reactions were started with an initial denaturation step at 95°C for 5 min, followed by 45 cycles including denaturation step at 95°C for 10s, extension step at 72°C for 10s and primer annealing step for 10s at varying temperatures: 62°C for K103N (AAC/AAT), 60°C for Y181C, 48°C for K70R and 56°C for M184V, T215Y and T215F. Specific PCR amplification was checked by thermal denaturation analysis of PCR products. All ASPCR assays were run in duplicate, and the arithmetic mean was used for further analysis. Accuracy and precision of ASPCR assays were calculated as previously described [1].

*Quantification of drug-resistant HIV-1 variants in clinical samples*

The baseline sample collected before intake of the first AZT dose was supposed to contain HIV-1 wild type only, since prevalences of reverse transcriptase mutations associated with drug resistance among treatment-naïve HIV-1-infected pregnant Tanzanian women was shown to be <5% [8,9]. Furthermore, we analyzed all clinical baseline samples by population-based sequencing and did not find any RT mutation (data not shown). The baseline sample was used to calculate the individual threshold, which compensates for individual sequence variability in the primer-binding site [1]. The individual cut-off for each woman, indicating the presence of drug-resistant virus, was set to the mean ΔCP of the baseline sample minus three standard deviations of 100% wild-type DNA control. For calculating ΔΔCP, delivery and postnatal samples were correlated to the respective baseline sample (ΔCP of baseline sample minus ΔCP of follow-up sample). Newborns’ samples were related to the respective maternal baseline sample. Subsequently, the proportion of drug-resistant HIV-1 was inferred from standard curves obtained by the ΔΔCP values.

As it is known that polymorphisms at primer binding sites have profound effects on the discriminatory ability of ASPCR assays [1,10], samples exhibiting an abnormally high CP value (CP>14) with the non-selective primer were excluded from the analysis. All serial specimens from one patient were tested in the same experimental run. Each run was performed with a set of mutant and wild-type DNA standards.

**References S**

1. Hauser A, Mugenyi K, Kabasinguzi R, Bluethgen K, Kuecherer C, et al. (2009) Detection and quantification of minor human immunodeficiency virus type 1 variants harboring K103N and Y181C resistance mutations in subtype A and D isolates by allele-specific real-time PCR. Antimicrob Agents Chemother 53: 2965–2973.

2. Primer X: Automated design of mutagenic primers for site-directed mutagenesis. Available: [www.bioinformatics.org/primerx](http://www.bioinformatics.org/primerx). Accessed 7 September 2011.

3. Walter H, Schmidt B, Korn K, Vandamme AM, Harrer T, et al. (1999) Rapid, phenotypic HIV-1 drug sensitivity assay for protease and reverse transcriptase inhibitors. J Clin Virol 13: 71–80.

4. Los Alamos National Laboratory: HIV Sequence Database. Sequence Search Interface. Available: http://www.hiv.lanl.gov/components/sequence/HIV/search/search.html. Accessed 7 September 2011.

5. Arroyo MA, Hoelscher M, Sateren W, Samky E, Maboko L, et al. (2005) HIV-1 diversity and prevalence differ between urban and rural areas in the Mbeya region of Tanzania. AIDS 19: 1517–1524.

6. Hoelscher M, Kim B, Maboko L, Mhalu F, von Sonnenburg F, et al. (2001) High proportion of unrelated HIV-1 intersubtype recombinants in the Mbeya region of southwest Tanzania. AIDS 15: 1461–1470.

7. Arroyo MA, Hoelscher M, Sanders-Buell E, Herbinger KH, Samky E, et al. (2004) HIV type 1 subtypes among blood donors in the Mbeya region of southwest Tanzania. AIDS Res Hum Retroviruses 20: 895–901.

8. Nyombi BM, [Holm-Hansen C](http://www.ncbi.nlm.nih.gov/pubmed?term="Holm-Hansen C"%5BAuthor%5D), [Kristiansen KI](http://www.ncbi.nlm.nih.gov/pubmed?term="Kristiansen KI"%5BAuthor%5D), [Bjune G](http://www.ncbi.nlm.nih.gov/pubmed?term="Bjune G"%5BAuthor%5D), [Müller F](http://www.ncbi.nlm.nih.gov/pubmed?term="Müller F"%5BAuthor%5D). (2008) Prevalence of reverse transcriptase and protease mutations associated with antiretroviral drug resistance among drug-naïve HIV-1 infected pregnant women in Kagera and Kilimanjaro regions, Tanzania. [AIDS Res Ther](http://www.ncbi.nlm.nih.gov/pubmed/18570675) 5: 13.

9. Somi GR, Kibuka T, Diallo K, Tuhuma T, Bennett DE, et al. (2008) Surveillance of transmitted HIV drug resistance among women attending antenatal clinics in Dar es Salaam, Tanzania. Antivir Ther 13 Suppl 2: 77–82.

10. Paredes R, Marconi VC, Campbell TB, Kuritzkes DR (2007) Systematic evaluation of allele-specific real-time PCR for the detection of minor HIV-1 variants with pol and env resistance mutations. J Virol Methods 146: 136–146.
